# Combined Bone Transportation and Lengthening Techniques for the Treatment of Septic Nonunion of the Forearm Followed by Tendon Transfer

**DOI:** 10.1155/2017/9672126

**Published:** 2017-07-20

**Authors:** Konstantinos Ditsios, Eirini Iosifidou, Lazaros Kostretzis, Panagiotis Konstantinou, Iosafat Pinto, Ioannis Theodoroudis, Ippokratis Hatzokos

**Affiliations:** ^1^First Department in Orthopaedics and Trauma Surgery, Georgios Papanikolaou Hospital, Aristotle University of Thessaloniki, Thessaloniki, Greece; ^2^Department in Orthopaedics and Trauma Surgery, Agios Pavlos General Hospital, Thessaloniki, Greece; ^3^Second Department in Orthopaedics and Trauma Surgery, G. Gennimatas General Hospital, Aristotle University of Thessaloniki, Thessaloniki, Greece

## Abstract

Infected nonunion of a forearm fracture complicated by a considerable skin-muscle defect poses a great challenge to orthopaedic surgeons. The treatment strategy comprises eradication of the infection, ensuring bony union and soft tissue coverage along with functional restoration. We report a case of a 23-year-old man with an open Gustilo-Anderson IIIb fracture complicated by infected nonunion after internal fixation. After thorough surgical debridement, a considerable soft tissue defect, extensor muscle loss, and posterior interosseous nerve laceration had to be addressed. He was finally treated with bone transportation and bone lengthening followed by tendon transfers.

## 1. Introduction

With the use of LCP plates and locking screws, failure of the internal fixation of forearm fractures is rare. Usually immediate plate fixation of an open fracture of the forearm grants a very low rate of infection and nonunion [1].

The presence of infection combined with bone loss and extensive devascularization creates an unsuitable environment for internal fixation as well as proper bone healing. Furthermore, nonunion of forearm diaphysis fractures requires anatomic restoration due to the fact that any nonanatomic deviance reflects directly to the function of the adjacent joints.

Priorities in this complex setting are eradication of the infection, obtainment of clean, adequate soft tissue envelope, and restoration of skeletal stability and functional anatomy. Any muscular defects of nerve palsies have to be dealt with at a later stage with tendon transfers.

## 2. Case Report

We report a case of a 23-year-old right-handed man who was admitted to our department after a car accident, with an open fracture of his left forearm (IIIb in the Gustilo-Anderson classification), extensor muscular mass loss, and a posterior interosseous nerve laceration. No other neurovascular injuries were present.

**Figure 1:**
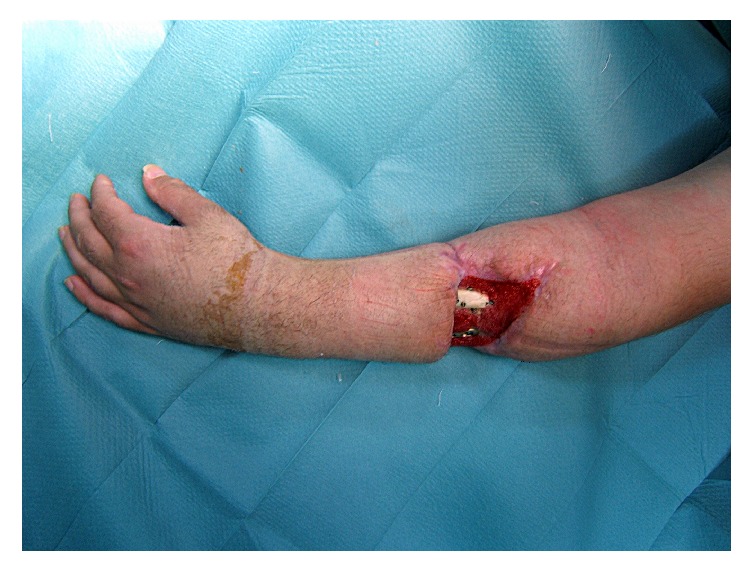
After thorough surgical debridement, a considerable soft tissue defect was created and bone was left exposed.

The treatment on his admission was debridement and internal fixation of the ulnar and radial fractures with LCP plates [1, 2]. Five months later, the patient developed deep infection with tissue necrosis in the trauma area. The patient was on empirical antistaphylococcal antibiotics and a reliable culture could not be obtained. Surgical debridement resulted in a soft tissue defect. We used vacuum assisted closure system (VAC, KCI Kinetic Concepts Inc., San Antonio, TX) combined with administration of culture specific antibiotics to resolve the problems of infection and poor bone coverage (Figure 1).

One month later, a new operation was carried out. Two unilateral external fixators (Orthofix, EBI Medical Systems, Parsippany, NJ) were applied to both radius and ulna. The fixation plates were removed and all devascularized tissues were excised to healthy, bleeding borders.

The meticulous debridement left two extensive osseous defects of the radius and ulna (radius: 8 cm; ulna: 4 cm) combined with a serious soft tissue defect (4 cm in diameter). The forearm was acutely shortened by 4 cm in order to facilitate adequate soft tissue coverage. A radial osteotomy was performed distally to the defect so as to treat the remaining defect of 4 cm by segmental bone transportation.

**Figure 2:**
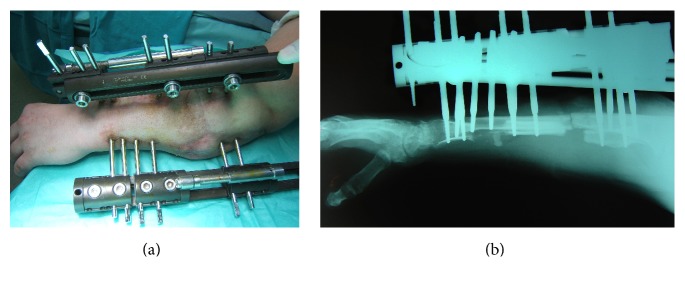
One external fixator was applied to each bone, radius, and ulna. They were used for segmental bone transportation of the radius and later distraction osteogenesis of both radius and ulna.

Transportation of the radial bone segment commenced after a week at a rate of 0.25 mm/6 hours and it was completed two months later. The docking site was freshened and grafted with iliac crest bone, demineralized bone matrix (DBM), and concentrated autologous bone marrow. An ulnar osteotomy was performed at the same surgical procedure, because the ulna had already been healed and distraction osteogenesis of both radius and ulna commenced after a week at the same rate of 0.25 mm/6 hours to restore the length of the forearm (Figure 2).

The external fixator of the ulna remained for 12 months, while the external fixation of the radius was removed at 13.5 months, when the docking site has been healed and consolidation of the regenerated bone was evident on three out of four cortices on anteroposterior and lateral radiographs. The regenerated bone of the radius was slightly palmary angulated (~10°) after the removal of the frame. During the lengthening process, the patient wore a volar forearm splint to help prevent contracture of the wrist. Active range-of-motion exercises were encouraged for the ipsilateral elbow and shoulder and passive range-of-motion exercises for the fingers, three weeks after commencing bone transportation.

One and a half year after the second operation, a tendon transfer was performed in order to improve the lost hand function. We used flexor carpi radialis (FCR) to the extensor carpi radialis brevis (ECRB) to restore wrist extension, because pronator teres (PT) and all the extensor muscles were destroyed during the car accident [3].

The patient did not have a palmaris longus and for the extension of the digits we used the flexor digitorum superficialis of the ring finger (FDS) tendon to extensor digitorum communis (EDC) [3].

**Figure 3:**
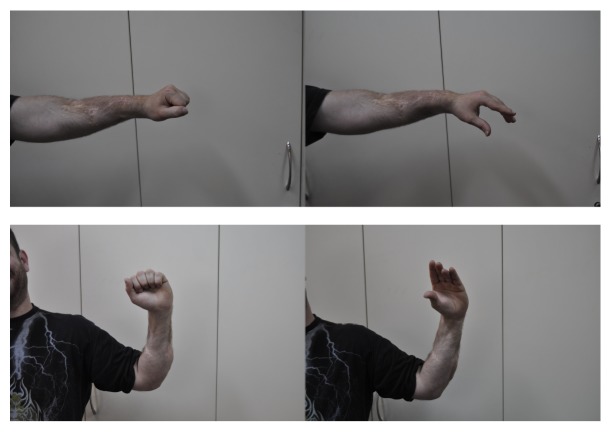
Two years after the final operation. The surgical wound has healed uneventfully. The patient can extend his wrist and fingers, and the thumb is out of the palm.

Finally, we used Z lengthening of the flexor pollicis longus and transferred the FDS tendon of the ring finger to the extensor pollicis longus. Two years later, the patient had full function of his elbow (extension/flexion: 0/145°), wrist flexion 50°, extension 0°, pronation 65°, supination 25°, full extension of the fingers, and the thumb out of the palm. The grip strength was 80% of the contralateral side and he reported no pain on daily activities (Figure 3). No complications were noted at the follow-up examinations.

## 3. Discussion

Injuries to the forearm, complicated with septic nonunion and skin-muscular mass defects, are rare [2]. However, the treatment of this condition in the forearm is a great challenge to orthopaedic surgeons because it leads to functional impairment.

The most common treatment techniques when dealing with bone loss are autogenous bone grafting and the use of nonvascularized fibular graft but their use is limited by a bone loss size of less than 6 cm [4, 5]. For larger defects, the use of vascularized fibular graft has been proposed with good results. Nonetheless, it requires a high degree of expertise and is often complicated by donor-site morbidity [6].

In our case, the presence of infection makes grafting risky. In these circumstances, different treatment modalities have been proposed, including radioulnar fusion [7], the technique of induced membrane described by Masquelet [8], and the techniques of distraction osteogenesis. Bone transportation and lengthening techniques are the most versatile techniques, allowing for precise restoration of the length loss with less invasion [9, 10]. Due to the long duration of the treatment, the psychological profile and the patient's willingness are among key prerequisites to the process of tissue histogenesis.

Many authors have reported good to excellent results with these techniques in the treatment of osseous defects that resulted from traumatic cases and septic nonunions [9–12]. When reviewing the literature regarding segmental defects in the tibia, the Ilizarov techniques have better results and fewer complications than bone grafting [13].

We describe our case of successful treatment of a substantial osseus and soft tissue defect of the forearm caused by septic nonunion. We used bone transportation and lengthening techniques and after 13.5 months we managed to restore the defect and achieve union. After ensuring the eradication of the infection, we used tendon transfer principles to restore the function of the hand.

## 4. Conclusion

Combined bone transportation and lengthening techniques using the unilateral external fixator followed by tendon transfers are a good choice for complex reconstructive cases in the forearm.
